# The hidden impact of myopia severity on interocular suppression in myopia: a cross-sectional study

**DOI:** 10.3389/fmed.2025.1481541

**Published:** 2025-06-19

**Authors:** Yan Luo, Xiyang Yang, Enwei Lin, Min Kong, Wuqiang Luo, Qi Chen, Jin Zeng, Li Yan, Lili Li, Xin Xiao

**Affiliations:** ^1^Visual Science and Optometry Center, The People's Hospital of Guangxi Zhuang Autonomous Region, Nanning, China; ^2^Department of Ophthalmology, Guangdong Provincial People’s Hospital (Guangdong Academy of Medical Sciences), Southern Medical University, Guangzhou, China; ^3^National Engineering Research Center for Healthcare Devices, Guangzhou, China; ^4^Guangxi Key Laboratory of Eye Health, The People's Hospital of Guangxi Zhuang Autonomous Region, Nanning, China; ^5^Department of Scientific Research, The People's Hospital of Guangxi Zhuang Autonomous Region, Nanning, China

**Keywords:** myopia, interocular suppression, spherical equivalent (SE), association, dose–response relationship, cross-sectional study

## Abstract

**Objective:**

This study aimed to investigate the association between spherical equivalent (SE) and interocular suppression in myopic adults, addressing the knowledge gap in functional visual impairments beyond structural changes.

**Methods:**

This hospital-based cross-sectional study included 988 myopic patients (aged 18.0–48.7 years, SE ≥ 0.50D). Grating stereopsis (GS), fine stereopsis at 1.5 m (FS1.5), fine stereopsis at 0.8 m (FS0.8), division, fusion, and interocular suppression were examined via computer-based tasks. Multivariate logistic regression analysis and restricted cubic splines (RCSs) were used to analyze the dose–response relationships between SE and the prevalence of suppression disorders (permanent suppression or binocular rivalry suppression). Sensitivity analysis and subgroup analysis were used.

**Results:**

The prevalence of suppression disorders was 30.6%. Multivariate logistic regression analysis revealed a dose–response relationship between SE and the prevalence of suppression disorder (odds ratio [OR]: 1.08, 95% CI: 1.00–1.17, *p* = 0.044) after adjusting for age, sex, anisometropia, cylindrical anisometropia, division, fusion, best corrected visual acuity (BCVA), FS0.8, FS1.5, and GS. Restricted cubic splines analysis revealed that the odds ratio of suppression disorder increased approximately linearly with the increase in spherical equivalent (*P* for non-linearity = 0.7633 > 0.05). Subgroup analyses showed that this association persisted in those aged <25 years (OR: 1.15; 95% CI: 1.04 ~ 1.27, *p* = 0.006), those with normal GS (OR: 1.17, 95% CI, 1.03–1.34, *p* = 0.020), and those with normal FS0.8 (1.09, 95% CI: 1.01–1.18, *p* = 0.026). In a sensitivity analysis that categorized myopia into three groups, a statistically significant positive association between high myopia (OR: 1.87, 95% CI: 1.10–3.29, *p* = 0.025), moderate myopia (OR: 1.75, 95% CI: 1.04–3.03, *p* = 0.039), and suppression disorder was found after adjustment for covariates.

**Conclusion:**

Myopia severity independently correlates with suppression disorders, suggesting the need for functional vision screening and personalized myopia correction strategies in high-risk populations.

## 1 Introduction

Myopia has become more common in recent decades. By 2050, it is predicted that 50% of the population will have myopia and that 10% will have high myopia (1). Myopia can reduce monocular or binocular visual acuity and also impair binocular vision function, such as stereopsis (2) and binocular rivalry (3). Interocular suppression is also a common abnormality in binocular visual function, especially for binocular rivalry (4). Interocular suppression involves complex neural mechanisms, where the brain prioritizes information from one eye while inhibiting the other when the two eyes receive conflicting information. This mechanism is crucial for maintaining visual stability, avoiding binocular visual conflict, and enhancing visual processing efficiency (5). Imbalances in this mechanism can lead to visual disorders such as amblyopia and strabismus (6), affecting stereopsis and depth perception.

Recent research has shown a link between anisometropia and interocular suppression, particularly in individuals with anisometropic amblyopia (7). This can affect their ability to cooperate with binoculars and manage visual information conflicts (8–10). However, most current research has focused on amblyopic patients, with relatively few studies examining interocular suppression in a population with normal vision. Our previous research revealed an intermittent suppression phenomenon in the majority of the normal population, who did not have eye diseases and had normal or corrected vision (11). Additionally, our team reported that individuals with myopia presented increased interocular suppression compared with those with normal vision when exposed to low-and high-frequency temporal frequency stimulation (12, 13). However, there are still some gaps in current research due to limited sample sizes, variations in detection methods, and a lack of studies focusing on adult myopic patients with varying degrees of myopia.

Therefore, this study aims to systematically evaluate the dose–response relationship between the equivalent spherical lens (SE) and interocular suppression in myopic patients for the first time through a large-scale cross-sectional investigation and to analyze its interaction with stereopsis, division, and fusion functions. These findings will not only deepen the understanding of visual function impairments in myopic patients but also provide a scientific basis for developing functional vision screening and personalized myopia correction strategies.

## 2 Methods

### 2.1 Participants

We consecutively enrolled 988 Chinese myopic adults from the Optometry Clinic of People’s Hospital of Guangxi between 1 March and 30 September 2022. The inclusion criteria for participants were as follows: (1) aged 18–48 years; (2) SE ≥ 0.50 D in at least one eye, myopic anisometropia of SE no more than 2.00 D; and (3) best-corrected visual acuity (BCVA) < 0.1 logMAR (Snellen>20/25).

The exclusion criteria were as follows: (1) unable to cooperate with the examination; (2) had a history of eye surgery; (3) had binocular alignment or motor dysfunction, such as strabismus or nystagmus; (4) had any other eye diseases except myopia; and (5) had a history of systemic diseases, such as heart, liver and kidney, and mental diseases. This study conformed to the Declaration of Helsinki and was reviewed and approved by the Ethics Committee of People’s Hospital of Guangxi (Approval No. KY-KJT-2022-047). All patients provided written informed consent to participate in the study and for their data to be published.

### 2.2 Measurement

#### 2.2.1 Measurement of visual acuity

Visual acuity was examined via an E-letter standard logarithmic visual acuity chart (SJ-LED-01, Guangzhou Shijia Medical Corporation, China) at 5 m. SE was determined by subjective refraction, and the best corrected visual acuity (BCVA) was recorded via the LogMAR value.

#### 2.2.2 Measurement of visual function

**Figure 1:**
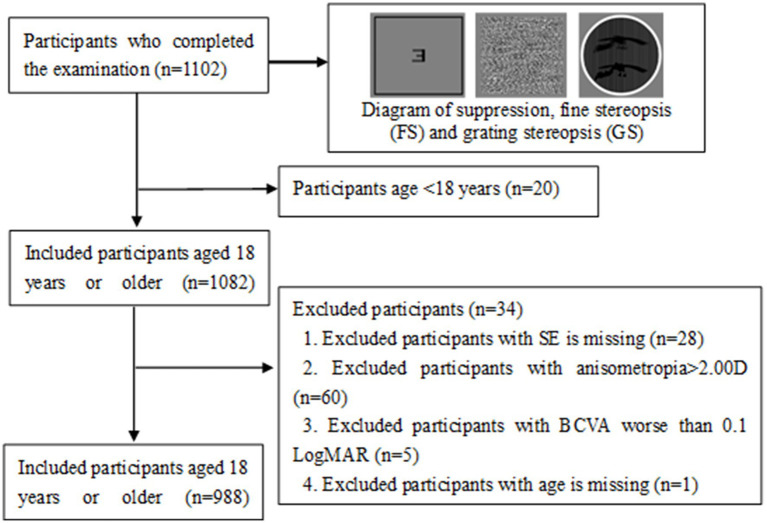
Flow chart of this study. FS, fine stereopsis; GS, grating stereopsis.

Adult myopia patients completed the computer-based tasks in a single assessment session, which was conducted in a quiet, dimly lit room. All tests were performed with refractive correction. The assessments of visual binocular function, including suppression (11, 14), grating stereopsis (GS) (15), fine stereopsis at 0.8 m (FS0.8), fine stereopsis at 1.5 m (FS1.5), division, and fusion, were developed by the National Engineering Research Center for Healthcare Devices (Figure 1). Stimulus templates were created via MATLAB and displayed on an LGD2343P 3D monitor with a resolution of 1980 × 1,080 and a refresh rate of 120 Hz. All tests were conducted at a constant room luminance with incandescent lamps (illuminance of 263 lx). All patients were adapted to this light level for 5 min and wore the polarized glasses with refractive correction. The distance was divided into near distance and moderate distance. All examinations were performed by the same skilled operator and repeated at least 3 times to obtain average data.

#### 2.2.3 Measurement of interocular suppression

The interocular suppression test was developed on the basis of a previously described binocular integrated model (11, 16, 17). The stimulation parameters included presenting a letter distribution contour on a gray background (44 cd/m^2^) with a viewing angle of 38° × 18°. The center of the stimulation diagram featured an F-target (0.66° × 0.66°) for one eye and an L-target (0.66° × 0.66°) for the other eye. The patient used a dichoptic mirror to determine if both eyes could see the combined E-target.

#### 2.2.4 Measurement of grating stereopsis and fine stereopsis

Grating Stereopsis Program Parameters. The stimulation parameters included displaying a sinusoidal grating stimulus on a gray background (44 cd/m^2^) within a circular visual field of 10°. The spatial frequency of the grating was 2.38 cpd, with a contrast set at 80%. The grating moved to the right at a speed of 2 deg./s. Each grating stimulus was presented for 500 ms. Two bird targets (7.5° × 7.5°) appeared at the center of the grating background. Various levels of disparity, including 400″, 300″, 200″, and 100″ (stereoacuity was measured in seconds of arc), were used. The surrounding gratings served as a reference for relative non-parallax. The participants wore 3D polarized glasses and were instructed to identify the convex or concave status of the bird targets on the screen. The testing procedure followed the same steps as those described in the fine stereopsis process.

#### 2.2.5 Fine stereopsis program parameters

The stimulation parameters included displaying a random dot distribution map on a gray background (44 cd/m^2^) with a viewing area of 5° × 5°. Within this area, 1,250 random dots on a gray background of 250 cd/m^2^ were arranged. The participants observed a central E-target (3° × 3°) positioned in the center of the random dot distribution map. Various levels of disparity, including 400″, 300″, 200″, and 100″ (stereoacuity was measured in seconds of arc), were used. The surrounding random dots served as a reference for relative non-parallax. The participants wore 3D polarized glasses and were instructed to identify the orientation of the protruding E-target aperture on the screen by selecting the corresponding arrow icon via a mouse or keyboard. Initially, participants observed a protruding E-target of 400″ and were tasked with identifying the aperture orientation twice. Successful identification on both occasions led to the presentation of a smaller protruding E-target measuring 300″, with further reductions continuing down to 100″. If participants provided an incorrect response, the test reverted to the previous higher level of discrepancy. The final outcome was documented, with assessment distances categorized as near (0.8 m) or far (1.5 m).

### 2.3 Definition

Anisometropia was defined as the interocular difference in the myopic SE. Cylindrical anisometropia was defined as the difference in astigmatism between eyes. Various levels of grating stereopsis, including 400, 300, 200, and 100, were recorded using level 1, level 2, level 3, and level 4, respectively. Normal GS was defined as level 4, and abnormal GS was defined as levels 1 to 3. Various levels of near-fine stereopsis, including 400, 300, 200, and 100, were recorded as level 1, level 2, level 3, and level 4, respectively. Normal FS0.8 was defined as level 4, and abnormal FS0.8 was defined as levels 1 to 3. Similarly, various levels of distance fine stereopsis, including 400, 300, 200, and 100, were recorded at levels 1, 2, 3, and 4, respectively. Normal FS1.5 was defined as level 4, and abnormal FS1.5 was defined as levels 1 to 3. The results of interocular suppression were divided into three levels: normal, permanent suppression, and binocular rivalry suppression. Normal was defined as both eyes simultaneously seeing the E-target, which means that there was no interocular suppression. Permanent suppression was defined as both eyes seeing only the F-target or L-target (18). Binocular rivalry suppression was defined as both eyes seeing the F-target or L-target, or E-target alternatively (19). Suppression disorder was defined as the inability of both eyes to see the E-target simultaneously, including permanent suppression and binocular rivalry suppression.

### 2.4 Statistical analysis

All the statistical analyses were performed using R statistics software (version 4.3.1, The R Foundation, released on 2024-05-06). The normality of the distribution of the data was assessed via the Shapiro–Wilk test, and the data are presented as the means ± SDs for normally distributed data or medians (P_25_, P_75_) for non-normally distributed data. Correlation relationships were computed via a Pearson correlation test or the Spearman correlation test, depending on whether the data of interest were normally or non-normally distributed, respectively.

Multivariate logistic regression was used to determine the odds ratios (ORs) and 95% confidence intervals (CIs) for the relationship between SE and the prevalence of suppression disorders. In accordance with previous studies (3), covariates, including demographic variables (age and sex), interocular differences in SE and astigmatism (anisometropia and cylindrical anisometropia), and binocular function (division, fusion, and fine or grating stereopsis) were included in the adjusted model. Therefore, Model 1 was adjusted for age, sex, anisometropia, and cylindrical anisometropia. Model 2 was adjusted for division, fusion, and BCVA in addition to the variables from Model 1. Model 3 was adjusted for FS0.8, FS1.5, and GS, in addition to the variables from Model 2. To further explore the dose–response relationship between SE and suppression disorder, we employed a restricted cubic spline (RCS) to further analyze their non-linear relationship and plotted the RCS curve.

**Figure 2:**
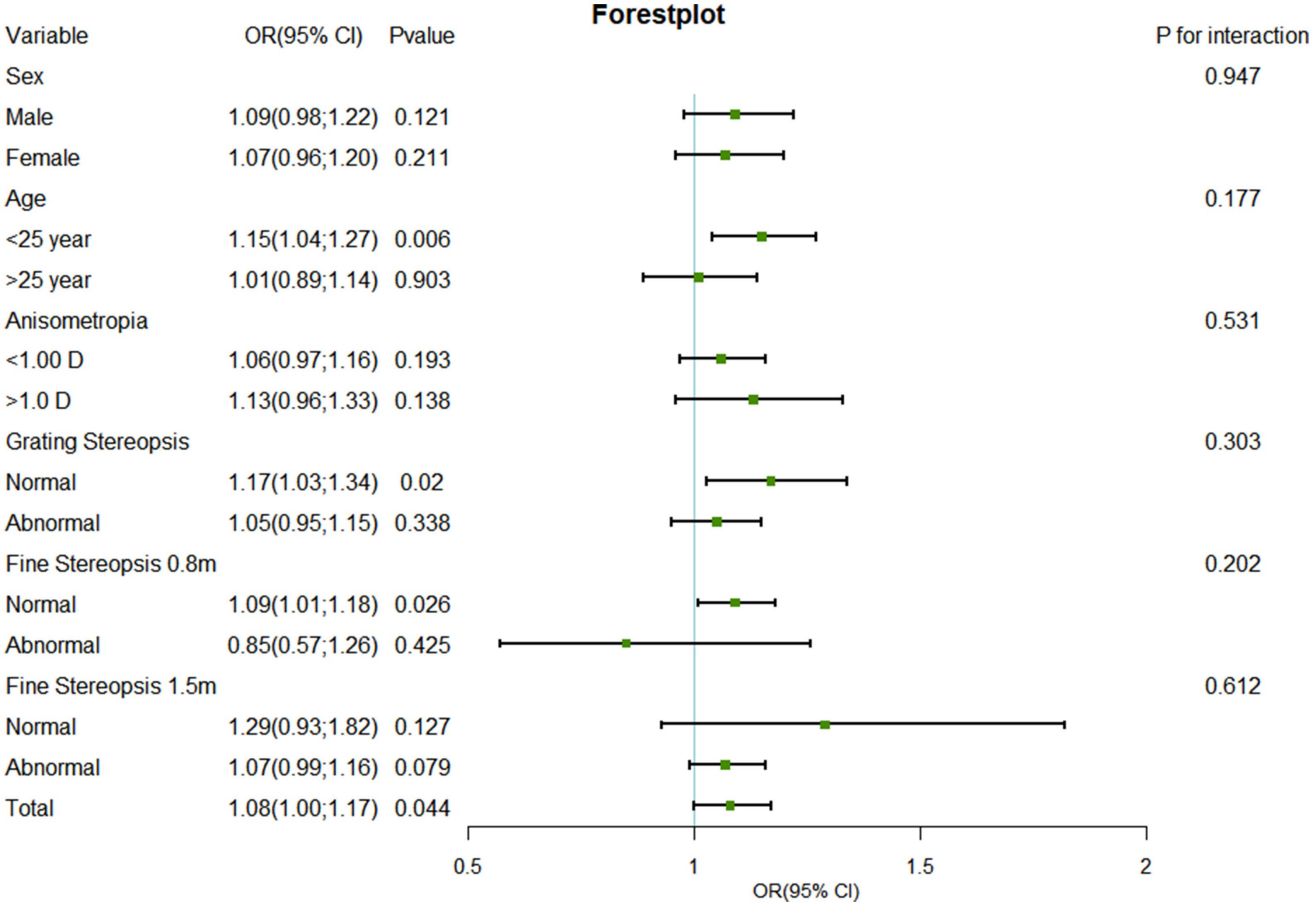
Forest plot of subgroup analysis of the association between SE and suppression disorder by covariate. SE, spherical equivalent; OR, odds ratio; CI, confidence interval.

To assess the robustness of our results, we performed several additional sensitivity analyses. We classified myopia participants into high myopia (≤ − 6.00 D), moderate myopia (≤ − 3.00 D and > − 6.00 D), and mild myopia (> − 3.00 D and <−0.50 D) groups on the basis of spherical equivalent, and calculated unadjusted and adjusted ORs and 95% confidence intervals (95% CIs) via univariate and multivariate logistic regression analysis (Table 1). In addition, we conducted a subgroup analysis via multivariate logistic regression analysis to explore the relationships among sex (male vs. female), age (≤25 years vs. >25 years), anisometropia (<1.00 D vs. ≥1.00 D), GS (normal vs. abnormal), FS0.8 (normal vs. abnormal), and FS1.5 (normal vs. abnormal). The *p*-values for the interactions among sex, age, anisometropia, GS, FS0.8, and FS1.5 were calculated, and a forest plot of the subgroup analysis results was drawn (Figure 2). *p* < 0.05 was considered statistically significant.

**Table 1:** Associations between SE and the risk of suppression disorders among myopia.

Myopia	No.	Unadjusted model		Model 1^a^		Model 2^b^		Model 3^c^	
		OR [95%CI]	*p*-value	OR [95%CI]	*p*-value	OR [95%CI]	*p*-value	OR [95%CI]	*p*-value
SE (Diopter)	988	1.09 [1.02;1.17]	0.013	1.09 [1.01;1.17]	0.018	1.09 [1.01;1.17]	0.020	1.08 [1.00;1.17]	0.044
Myopia group									
Mild myopia	119	1 (Ref.)		1 (Ref.)		1 (Ref.)		1 (Ref.)	
Moderate myopia	531	1.40 [0.89; 2.26]	0.152	1.48 [0.93; 2.41]	0. 104	1.49 [0.93; 2.44]	0.106	1.75 [1.04; 3.03]	0.039
High myopia	338	1.65 [1.03; 2.71]	0.040	1.69 [1.05; 2.79]	0.035	1.66 [1.02; 2.77]	0.045	1.87 [1.10; 3.29]	0.025

SE, spherical equivalent; BCVA, best corrected visual acuity; GS, grating stereopsis; FS0.8, fine stereopsis was measured at distances of 0.8 m; FS1.5, fine stereopsis was measured at distances of 1.5 m.

a Model 1 was adjusted for age, sex, anisometropia, and cylindrical anisometropia.

b Model 2 was adjusted for Model 1 + division, fusion, and BCVA.

c Model 3 was adjusted for Model 2 + FS0.8, FS1.5, and GS.

## 3 Results

### 3.1 Demographic and clinical characteristics of the subjects

**Figure 3:**
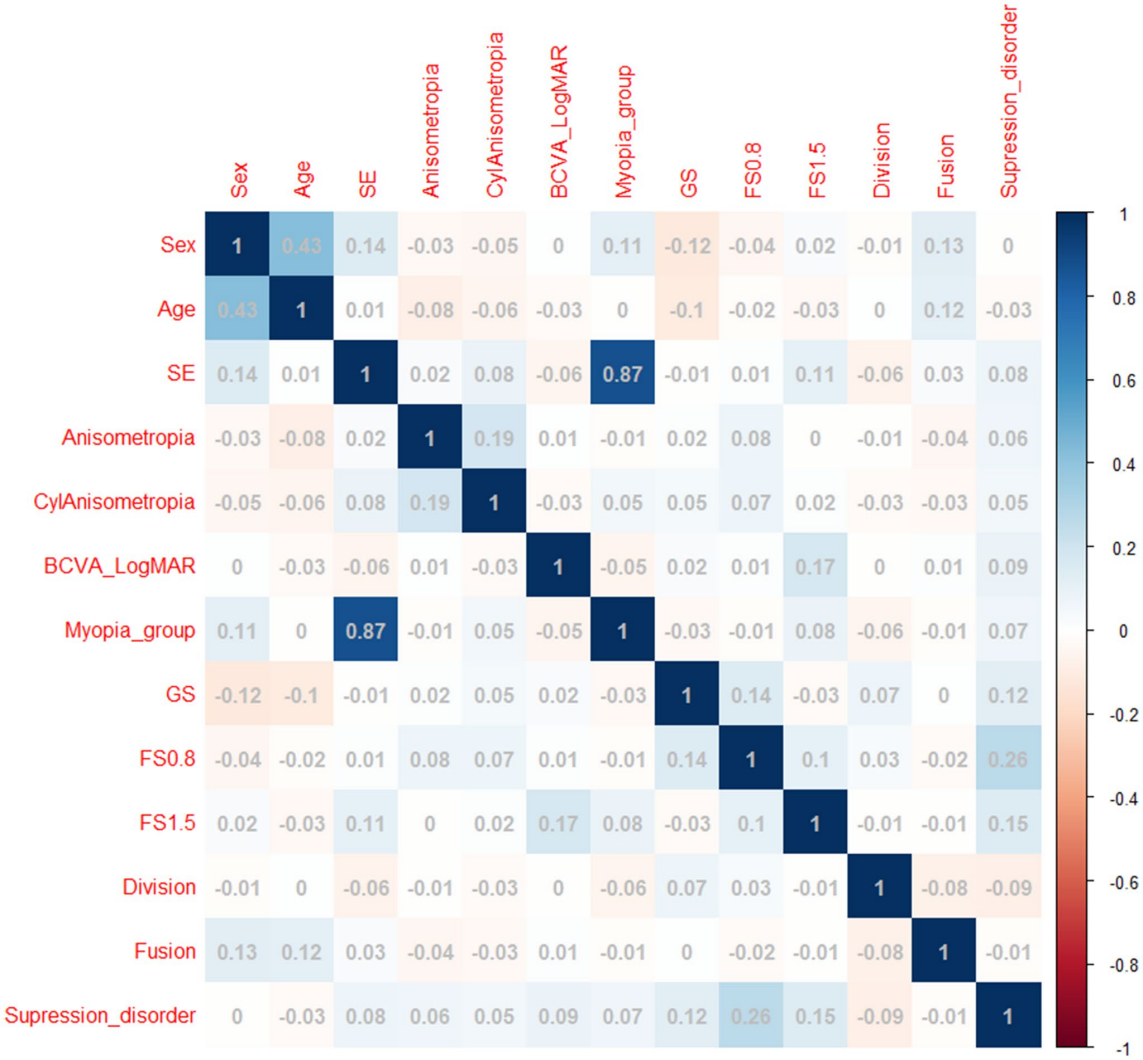
Heatmap of correlations between covariates and suppression disorders. SE, spherical equivalent; LogMAR, logarithm of the minimum angle of resolution; BCVA, best corrected visual acuity; CylAnisometropia, cylindrical anisometropia, interocular difference in astigmatism; FS0.8: fine stereopsis was measured at distances of 0.8 m; FS1.5: fine stereopsis was measured at distances of 1.5 m.

Between 1 March and 30 September 2022, a total of 1,102 Chinese adults with myopia were recruited, and 988 patients, aged 18.0–48.7 years, completed the test at the Optometry Clinic of People’s Hospital of Guangxi. The flow chart of this study is presented in Figure 1. The baseline characteristics of the participants in the myopia group are presented in Table 2. There were statistically significant differences in sex, age, SE, anisometropia, and FS1.5 among the mild myopia, moderate myopia, and high myopia groups (all *p* < 0.05). The correlation heatmap also revealed similar results (Figure 3). Suppression disorders were significantly positively associated with FS0.8 (*r* = 0.26, *p* < 0.001), FS1.5 (*r* = 0.15, *p* < 0.001), and GS (*r* = 0.12, *p* < 0.001), respectively.

**Table 2:** Characteristics of the participants by myopia group.

Variables	Total (*n* = 988)	Mild myopia (*n* = 119)	Moderate myopia (*n* = 531)	High myopia (*n* = 338)	*p*-value
Sex					<0.001
Male	468 (47.4%)	83 (69.7%)	234 (44.1%)	151 (44.7%)	
Female	520 (52.6%)	36 (30.3%)	297 (55.9%)	187 (55.3%)	
Age (year)	25.4 (6.18)	23.6 (5.86)	26.2 (6.37)	24.7 (5.79)	<0.001
SE (D)	5.18 (1.97)	2.14 (0.58)	4.48 (0.85)	7.34 (1.18)	<0.001
Anisometropia (D)	0.51 (0.46)	0.60 (0.56)	0.48 (0.44)	0.54 (0.45)	0.018
Cylindrical anisometropia (D)	0.32 (0.34)	0.30 (0.30)	0.32 (0.34)	0.35 (0.34)	0.265
BCVA (LogMAR)	−0.01 (0.03)	0.00 (0.02)	0.00 (0.03)	−0.01 (0.03)	0.289
GS					0.577
Normal	384 (42.2%)	42 (37.8%)	207 (42.3%)	135 (43.5%)	
Abnormal	526 (57.8%)	69 (62.2%)	282 (57.7%)	175 (56.5%)	
FS0.8:					0.443
Normal	935 (94.6%)	110 (92.4%)	506 (95.3%)	319 (94.4%)	
Abnormal	53 (5.36%)	9 (7.56%)	25 (4.71%)	19 (5.62%)	
FS1.5:					0.015
Normal	154 (15.6%)	22 (18.5%)	95 (17.9%)	37 (10.9%)	
Abnormal	834 (84.4%)	97 (81.5%)	436 (82.1%)	301 (89.1%)	
Division	−7.56 (5.91)	−6.98 (4.90)	−7.43 (5.00)	−7.98 (7.40)	0.227
Fusion	4.02 (4.89)	3.77 (3.61)	4.21 (5.21)	3.79 (4.76)	0.411
Suppression disorder					0.110
No	686 (69.4%)	91 (76.5%)	371 (69.9%)	224 (66.3%)	
Yes	302 (30.6%)	28 (23.5%)	160 (30.1%)	114 (33.7%)	

LogMAR, logarithm of the minimum angle of resolution; BCVA, best corrected visual acuity; GS, grating stereopsis; FS0.8, fine stereopsis was measured at distances of 0.8 m; FS1.5, fine stereopsis was measured at distances of 1.5 m.

### 3.2 Prevalence of suppression disorder and variation with covariates

**Figure 4:**
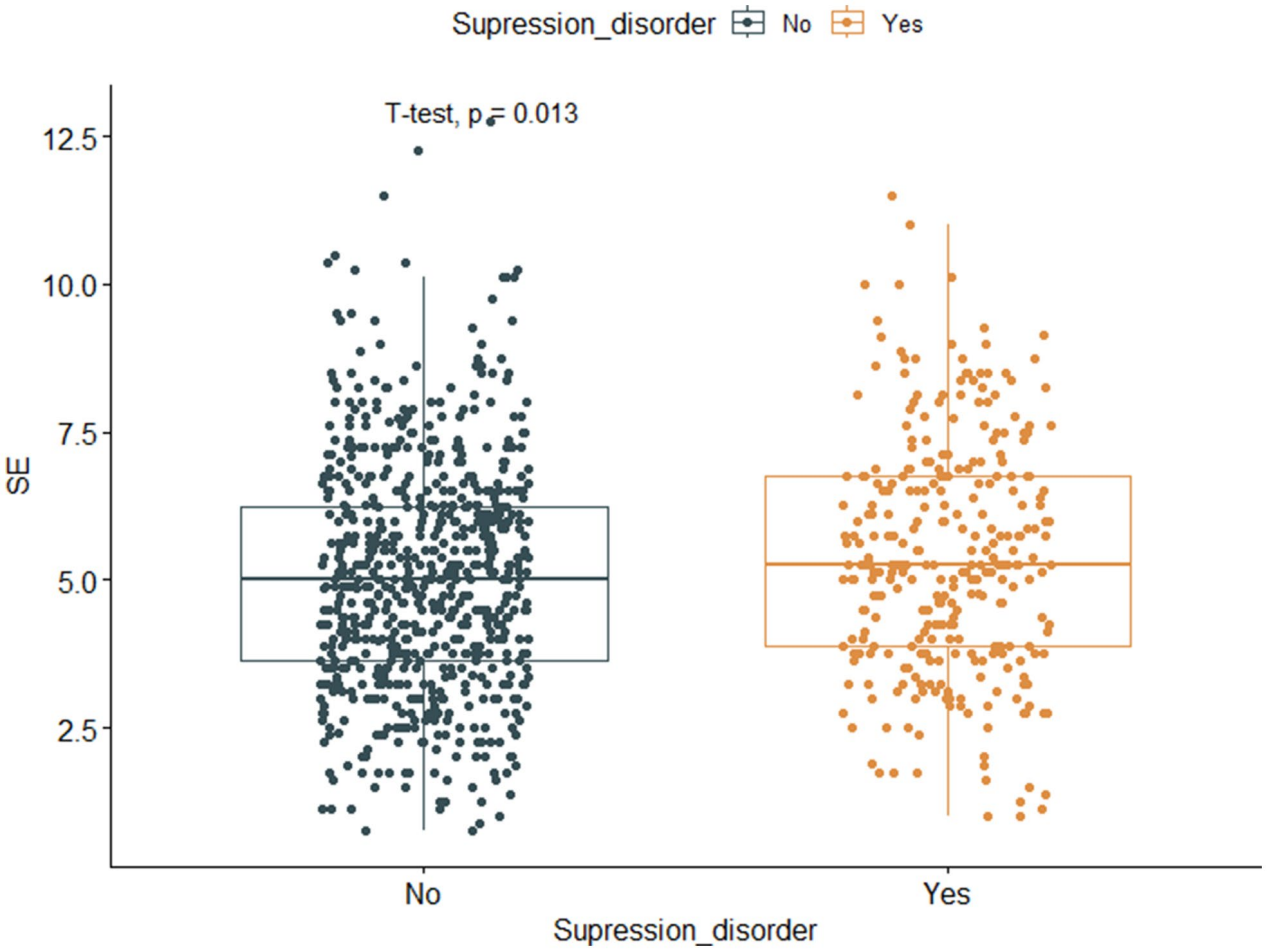
Boxplot and scatter plot of SE against suppression disorder. The horizontal axis represents suppression disorder (No, Normal, Yes, Abnormal), whereas the vertical axis represents myopic spherical equivalent. SE, spherical equivalent.

Among the 988 myopic participants, the prevalence of suppression disorders (permanent suppression or binocular rivalry suppression) was 30.6%, and the prevalence rates of mild myopia, moderate myopia, and high myopia were 23.5, 30.1, and 33.7%, respectively. Figure 4 shows that the SE of participants with suppression disorders was greater than that of participants without suppression disorders (*p* = 0.013). The associations between the covariates and the risk of suppression order are displayed in Table 3. Univariate analysis revealed that SE, BCVA, GS, FS0.8, FS1.5, and division were associated with suppression disorders (all *p* < 0.05).

**Table 3:** Associations between covariates and the risk of suppression disorder.

Variables	Normal (*n* = 686)	Abnormal (*n* = 302)	OR [95% CI]	*p*-value
Gender				0.951
Male	324 (47.2%)	144 (47.7%)	Ref.	
Female	362 (52.8%)	158 (52.3%)	0.98 [0.75;1.29]	
Age (year)	25.5 (6.20)	25.1 (6.14)	0.99 [0.97;1.01]	0.419
SE (D)	5.07 (1.96)	5.41 (1.97)	1.09 [1.02;1.17]	0.013
Anisometropia (D)	0.50 (0.45)	0.56 (0.47)	1.33 [0.99;1.77]	0.059
Cylindrical anisometropia (D)	0.31 (0.31)	0.35 (0.39)	1.37 [0.93;2.02]	0.147
BCVA (LogMAR)	−0.01 (0.03)	0.00 (0.02)	6,666 [10.6;4,183,144]	0.001
Myopia group				0.110
Mild myopia	91 (13.3%)	28 (9.27%)	Ref.	
Moderate myopia	371 (54.1%)	160 (53.0%)	1.40 [0.89;2.25]	
High myopia	224 (32.7%)	114 (37.7%)	1.65 [1.03;2.70]	
GS				<0.001
Normal	290 (46.3%)	94 (33.1%)	Ref.	
Abnormal	336 (53.7%)	190 (66.9%)	1.74 [1.30;2.34]	
FS0.8				<0.001
Normal	676 (98.5%)	259 (85.8%)	Ref.	
Abnormal	10 (1.46%)	43 (14.2%)	11.1 [5.68;23.7]	
FS1.5				<0.001
Normal	131 (19.1%)	23 (7.62%)	Ref.	
Abnormal	555 (80.9%)	279 (92.4%)	2.85 [1.82;4.65]	
Division	−7.23 (5.97)	−8.34 (5.71)	0.97 [0.95;0.99]	0.007
Fusion	4.05 (4.97)	3.94 (4.70)	1.00 [0.97;1.02]	0.740

LogMAR, logarithm of the minimum angle of resolution; SE, spherical equivalent; BCVA, best corrected visual acuity; GS, grating stereopsis; FS0.8, fine stereopsis was measured at distances of 0.8 m; FS1.5, fine stereopsis was measured at distances of 1.5 m.

### 3.3 Associations between SE and suppression disorders

A statistically significant positive association between SE and suppression disorder was observed after adjusting for potential confounders (Table 1). The adjusted OR of SE for suppression disorders was 1.08 (95% CI: 1.00–1.17, *p* = 0.044). Compared with those of participants with mild myopia, the adjusted ORs for suppression disorders in the moderate myopia and high myopia groups were 1.75 (95% CI: 1.04–3.03, *p* = 0.039) and 1.87 (95% CI: 1.10–3.29, *p* = 0.025), respectively.

**Figure 5:**
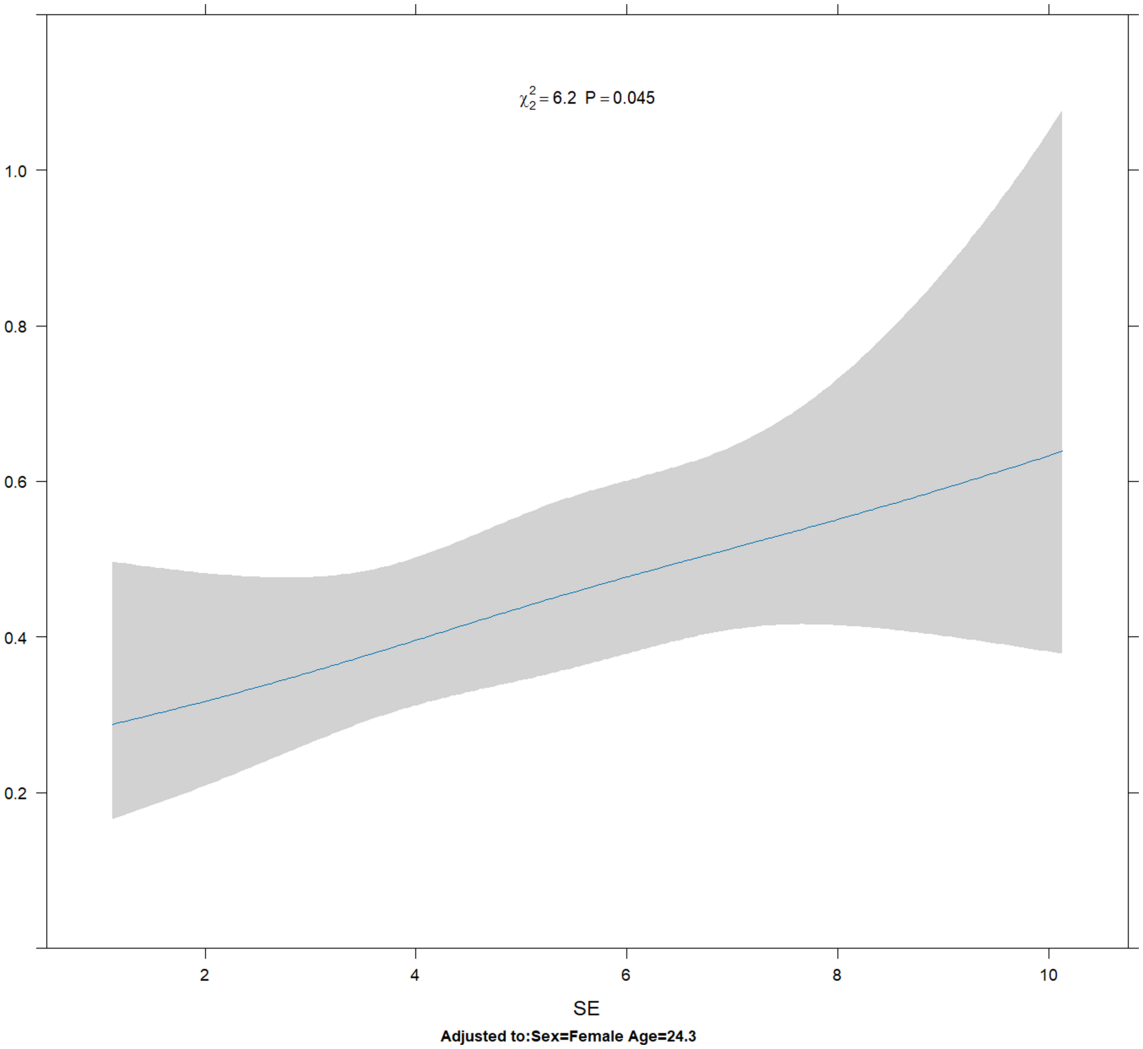
Association of SE with the risk of suppression disorders. SE, spherical equivalent.

The results of restricted cubic spline analysis revealed that the Akaike information criterion (AIC) values for 3 knots, 4 knots, and 5 knots were 1219.414, 1221.359, and 1223.327, respectively. The likelihood ratio test revealed that the overall *p*-value was statistically significant (*p* = 0.045 < 0.05), but the non-linear *p*-value was not statistically significant (*p* = 0.7633 > 0.05). The RCS curve revealed that the risk ratio of suppression disorder increased approximately linearly with the increase in spherical equivalent (Figure 5).

### 3.4 Stratified analyses based on additional variables

In several subgroups, stratified analysis was performed to assess potential effect modifications on the relationship between SE and suppression disorders. No significant interactions were found in any subgroup after stratification by sex, age, anisometropia, GS, FS0.8, or FS1.5 (Figure 2). SE was associated with the prevalence of suppression disorders among those aged ≤25 years (OR: 1.15, 95% CI: 1.04–1.27, *p* = 0.006), among those with a normal GS (OR: 1.17, 95% CI, 1.03–1.34, *p* = 0.020), and those with a normal FS0.8 (OR: 1.09, 95% CI, 1.01–1.18, *p* = 0.026).

## 4 Discussions

This novel study evaluated visual function and performed correlation analyses in myopic adults and revealed that interocular suppression (permanent suppression or binocular rivalry suppression) was more prevalent in individuals with high myopia or moderate myopia than in those with mild myopia. Consistent with our hypothesis, multivariate logistic regression and RCS analyses confirmed that the dose–response relationship between SE and suppression disorders addresses the knowledge gap regarding functional impairments in non-amblyopic myopia. The brain processes the selected input through various visual regions, including the primary visual cortex (V1) and the inferotemporal cortex (V2, V3, and V4), to resolve conflicts and maintain effective visual perception (9, 20). Interocular suppression occurs when the brain selectively inhibits input from one eye in response to conflicting visual information from both eyes (21). This selection was typically based on which eye provided more useful, clearer, or more stable information in the given context. Therefore, suppression is crucial in the visual system, especially when dealing with inconsistent visual information (22).

Our study revealed that even in the mild myopia group, 20–30% of patients exhibited interocular suppression. Our previous study revealed that intermittent monocular suppression, referred to as binocular imbalance, is common in the normal population (11). However, this intermittent monocular suppression differed from amblyopic suppression and was better defined as a non-suppressive event (5, 23, 24). This pattern of imbalance involves short cycles of suppression limited to 2–3 degrees of central vision, with one eye suppressed for 2–3 s, followed by binocular fusion and the other eye suppressed for 2–3 s before returning to binocular fusion (25). Regrettably, our study did not document the duration and pattern of interocular suppression, thus hindering its categorization as either transient (physiological phenomenon) or permanent (pathological phenomenon) suppression.

In the general myopic population, the effect of anisometropia on visual function is particularly pronounced (26–28). Anisometropia has the potential to create disparities in visual information processing between the eyes, increasing the likelihood of interocular suppression (29–31). Li et al. (32) identified a notable association between interocular suppression and visual function in individuals with anisometropic amblyopia (32). Nevertheless, even after controlling for potential confounding variables such as anisometropia, the spherical equivalent continued to emerge as a significant predictor of interocular suppression in adult myopic individuals, particularly within the high myopia subgroup. The physiological mechanisms of interocular suppression in myopic patients are intricate and multifactorial. These mechanisms include discrepancies in binocular visual input, poor synchronization of ocular accommodation, the accumulation of visual fatigue, and neural adaptation in the brain. The main reason for this was significant anisometropia (3), which led the brain to suppress the blurrier image while prioritizing the clearer image. As myopia progresses, functional impairments and accommodative lag become more pronounced (33), thereby increasing visual fatigue (34). Furthermore, the intrinsic minification and prism effects of the myopic spectacles could increase the brain’s load in processing visual signals. This could explain why the incidence of interocular suppression was greater in the high myopia group than in the mild and moderate myopia groups. Notably, prolonged and continuous monocular suppression affects visual function and increases myopia (35). Therefore, for myopic patients with permanent interocular suppression, refractive correction combined with visual function training may be considered. This approach may help patients improve visual quality and control myopia progression (36, 37).

In terms of physiological mechanisms, animal models indicate that prolonged monocular deprivation results in reduced excitatory drive in the deprived eye, leading to an imbalance in the activation of binocular cortical neurons (38). For many years, amblyopic defects have been explained with the assumption that amblyopia is an anatomically monocular deficiency and a lack of binocular vision function (6, 39). From this perspective, any residual binocular interactions were seen as purely suppressive, resulting in the loss of monocular (amblyopic eye) function. However, at present, more studies have suggested that suppression plays a major role in both binocular and monocular structural defects in amblyopic patients (39, 40). The weak and noisy visual signals from the amblyopic eye limit its visual function and are suppressed by the visual input from the other eye. Thus, amblyopia could be structurally binocular but functionally monocular, as the brain relies on the input from the better eye during natural viewing tasks (41). Stronger suppression is associated with more severe amblyopia and has been traditionally viewed as an adaptive mechanism to avoid diplopia. Conversely, it has been proposed that suppression may contribute to amblyopia, making it a potential target for treatment (42).

Hong et al. reported that the successful group presented lower levels of suppression than did those who did not improve in populations receiving standard amblyopia treatment (43). This result implies that patients with severe interocular suppression are more likely to experience poorer treatment outcomes. Our results revealed a significant positive association between impaired stereopsis and interocular suppression in myopic adults (44), which is in agreement with the findings of previous studies on children with amblyopia. This finding indicates that permanent suppression is often associated with other visual function impairments, which can affect the efficacy of amblyopia treatment. Consequently, in treating amblyopia, it is imperative to focus not only on improving visual acuity but also on establishing binocular visual function.

Interocular suppression impacts individuals of all ages, with its manifestation and consequences differing among individuals (45). Children and adolescents are frequently affected, particularly in instances of amblyopia or strabismus. The visual system in adults is relatively stable unless prolonged aberrant visual behaviors are present. With increasing age, some older adults might experience a decline in visual ability, including the onset or worsening of suppression, which could affect their independence and quality of life, such as reading difficulties and challenges in daily activities. In this study, we found that the significant positive association between suppression disorders and the spherical equivalent was more pronounced in individuals younger than 25 years old, possibly because of the heightened plasticity of the visual system in younger individuals, rendering them more vulnerable to alterations in the spherical equivalent.

Despite these significant associations, several limitations should be acknowledged. First, we did not detail the duration and type of interocular suppression, making it difficult to determine whether the observed suppression was physiological or pathological. Subsequent research efforts should aim to elucidate the relationship between varying levels of suppression and myopia by meticulously categorizing and quantifying the features of interocular suppression, thereby enhancing the understanding of its presentation in individuals with myopia. Second, the current study revealed a significant positive association between refractive error and interocular suppression. However, there was no comparison with a control group, and this association does not imply causation. To establish a causal relationship between refractive error and suppression, future longitudinal studies are warranted to investigate the lasting effects of spherical equivalent changes on suppression disorders and to examine individual variations compared with the control group. These advancements will enhance our understanding of the role of interocular suppression in myopia and offer a more evidence-based foundation for clinical interventions.

## 5 Conclusion

This study conducted a systematic evaluation of the association between the spherical equivalent and suppression disorders in adult myopic patients. We found that the prevalence of suppression disorders increased significantly with increasing myopia severity. This discovery offers novel perspectives on functional vision screening and personalized correction of clinical myopia, underscoring the imperative for additional research and practical implementation.

## Data Availability

The raw data supporting the conclusions of this article will be made available by the authors, without undue reservation.
